# Signs and symptoms of temporomandibular disorders and oral parafunctions in urban Saudi arabian adolescents: a research report

**DOI:** 10.1186/1746-160X-2-25

**Published:** 2006-08-16

**Authors:** Rabab M Feteih

**Affiliations:** 1 Department of Preventive Dental Sciences, Orthodontic Division, Faculty of Dentistry, King Abdulaziz University, P.O. Box 50790, Jeddah 21533, Saudi Arabia

## Abstract

**Background:**

The aim of this study was to evaluate the prevalence of signs and symptoms of temporomandibular disorders (TMD) and oral parafunction habits among Saudi adolescents in the permanent dentition stage.

**Methods:**

A total of 385 (230 females and 155 males) school children age 12–16, completed a questionnaire and were examined clinically. A stratified selection technique was used for schools allocation.

**Results:**

The results showed that 21.3% of the subjects exhibited at least one sign of TMD and females were generally more affected than males. Joint sounds were the most prevalent sign (13.5%) followed by restricted opening (4.7%) and opening deviation (3.9%). The amplitude of mouth opening, overbite taken into consideration, was 46.5 mm and 50.2 mm in females and males respectively. TMJ pain and muscle tenderness were rare (0.5%). Reported symptoms were 33%, headache being the most frequent symptom 22%, followed by pain during chewing 14% and hearing TMJ noises 8.7%. Difficulty during jaw opening and jaw locking were rare. Lip/cheek biting was the most common parafunction habit (41%) with females significantly more than males, followed by nail biting (29%). Bruxism and thumb sucking were only 7.4% and 7.8% respectively.

**Conclusion:**

The prevalence of TMD signs were 21.3% with joint sounds being the most prevalent sign. While TMD symptoms were found to be 33% as, with headache being the most prevalent. Among the oral parafunctions, lip/cheek biting was the most prevalent 41% followed by nail biting 29%.

## Background

Temporomandibular disorders have been recognized as a common orofacial pain condition. The American Dental Association in 1983 has suggested that the term Temporomandibular disorders (TMD) refers to a group of disorders characterized by: pain in the temporomandibular joint (TMJ), the periauricular area, or the muscles of mastication; TMJ noises (sounds) during mandibular function; and deviations or restriction in mandibular range of motion [1].

A number of epidemiological studies on the prevalence of TMD in children and adolescents have been published from different populations, where the prevalence of TMD varied from 9.8 to 80 percent (table 1). The lack of international standards, different kinds and qualities of examination methods play a role for the different estimations and reports on TMD [2-4].

**Table 1:** Prevalence (%) of signs and symptoms of TMD in different epidemiological studies on children and adolescents

**Investigator**	**Population**	**Subjects**				**Signs %**	**Symptoms %**
				
		**Sample Size**	**Females**	**Males**	**Age**		
Siebert 1975[45]	Germany	232	-	-	12–16	62–80	-
Grosfeld et al 1977[42]	Poland	250	133	117	13–15	68^*‡^	10
Williamson 1977[43]	US	304	175	129	6–16	35	-
Nilner 1981[17]	Sweden	440	218	222	7–14	64	36
		309	162	147	15–18	55	41
Egermark-Eriksson[25] 1982	Sweden	131	61	70	11	46	67
		135	59	76	15	61	74
Gazit et al 1984[30]	Israel	369	181	188	10–18	44^‡^	56
Ogura et al 1985[21]	Japan	2198	1103	1095	10–18	9.8*	
Dibbets 1985[24]	Holland	165	94	71	7–19	21	19
Grosfeld et al 1985[29]	Poland	400	203	197	15–18	68.2^‡^	-
Wanman et al 1986[33]	Sweden	285	139	146	17	56	20
Jamsa et al 1988[26]	Finland	109	-	-	10	41	-
		147	-	-	15	42	-
Motegi et al 1992[22]	Japan	7337	4118	3219	6–18	12.2	12.2
Keeling et al 1994[44]	USA	3428	-	-	6–12	10	-
Deng et al 1995[20]	China	634	326	308	12–15	21.9	-
		905	424	481	16–19	15.9	-
Abdel-Hakim 1996[38]	S. Arabia	330	136	194	14–21	-	32
Al Amoudi et al 1998[5]	S. Arabia	502	267	235	3–7	16.5	-
Farsi N. 1999[6]	S. Arabia	696	-	-	6–14	17.1	14.1
Thilander et al 2002[23]	Bogota	1441	756	685	13–17	25	11.4
Farsi N. 2003[7]	S. Arabia	734	420	314	12–15	20.2	31.1
Feteih 2005 ^†^	S. Arabia	385	230	155	12–16	21.3	34

* Signs + symptoms

^‡ ^Stethoscope was used

^† ^Present Study.

Few studies have been reported on the prevalence of TMD in Saudi Arabia in normal children during the primary [5], mixed [6] and permanent dentition [7] and adults [8]. Other Saudi reports were on signs and symptoms of TMD in specific patient and non patient subjects such as military students [9], female patients seeking orthodontic treatment [10], and dental students [11].

The prevalence of TMD is still not well known and more studies and comparisons are necessary to allow better understanding of the pathological aspects so as to address effective preventive and therapeutic measures.

The aim of this study was to use a cross sectional epidemiological study to investigate the prevalence of signs and symptoms of TMD in adolescent school children in the permanent dentition, males and females, through clinical examination and self reported questionnaire.

## Methods

The sample consisted of 430 school children, 255 females and 175 males, attending the seventh, eighth and ninth grade. Their age ranged from 12–16 years. To ensure random selection from the schools, using a stratified selection technique, six public schools were selected from different geographic locations in the city of Jeddah, in the western region of Saudi Arabia.

All students attending on the day of examination were included. Inclusion criteria were: all permanent dentition stage (absence of primary teeth), no history of orthodontic treatment, no craniofacial anomalies and all students should be Saudi nationals. Parents and children were informed regarding the purpose of the study and consent forms were obtained. Forty five students who did not fulfill the inclusion criteria or did not complete the questionnaire were excluded, and the final sample size was 385 students (230 females and 155 males).

### Clinical examination

The examination was carried out by two examiners from the department of Preventive Oral Sciences, an Orthodontist and a Paediatric Dentist. Inter and intra examiners calibration and standardization was done prior to the commencement of the study. Using Cohen's Kappa statistics, the reliability tests were 0.90 and 0.94 respectively. The examinations on the students were carried out in the schools under proper lighting and students were seated upright during the examination.

### Examination of TMD

#### 1. TMJ sound

Digital palpation of the TMJ was done using the middle and index fingers and by audibly listening during opening and closure of the mouth [12] and by palpation [13], no stethoscope was used. Listening to joint sounds was done with the examiner's ear within 5 cm of the TMJ as described by Goho and Jones [12]. The examiner placed the middle and index finger over the TMJ area on each side of the head and the student was asked to open and close several times. Any irregularities on closing or opening were recorded. This was again repeated with the little fingers pressed anteriorly in the external auditory meati, i.e. against the posterior aspect of the joint.

#### 2. Muscular disorder

Digital palpation of the TMJ and associated muscles was performed to detect tenderness using the index, middle and the third finger. The Masseter, Temporalis and the Sternocleidomastoid muscles were palpated bilaterally for tenderness according to the method of Vanderas [14]. Intraoral muscles were not examined. The TMJ tenderness was also assessed during mandibular movement according to the method of Dworkin [15]. Pain was registered as 'absent' or 'present'.

#### 3. Range of the mandibular motion

The amplitude of *maximum vertical opening *(MVO) was recorded using a Boley gauge according to Okeson [16]. The gauge was placed on the mandibular incisor edge adjacent to the midline. The child was asked to open as wide as possible and the inter-incisal distance measurement was recorded. This process was repeated twice, and the average was obtained. The overbite value was added to the measurement to obtain the MVO. In cases of openbite the inter-incisal distance was subtracted to obtain the exact MVO. The lower limit for normal mouth opening was considered 40 mm according to Okeson [16]. The *opening deviation *was defined as the displacement of mandible at least 2 mm to the right or left of an imaginary vertical line when the mandible had reached half of its vertical opening. The patient was asked to open the mouth slowly and this was repeated several times for confirmation [13].

### Questionnaire

The subjects and their parents were requested to answer a questionnaire that included history of frequent headache, jaw locking, hearing TMJ noises, difficulty opening the mouth and acute pain in the periauricular area during chewing. Other questions on parafunctional habits such as nail/check biting, bruxism, finger and thumb sucking were also included in the questionnaire.

### Data analysis

SPSS statistical package (ver.10) was used. The frequency and forms of appearances of TMD signs and symptoms were analyzed regarding the total number of subjects, for females and males separately. Comparisons were then carried out using Pearson's chi-square test. The level of significance was set at P < 0.05.

## Results

The prevalence of TMD signs and sex differences are shown in Table 2. In the whole sample, 21.3% had at least one sign of TMD. The least frequent sign was muscle tenderness (0.5%) while the most frequent sign was TMD sounds (13.5%).

**Table 2:** Percentage distribution of TMD signs according to gender

**TMD signs**	**Females n = 230%**	**Males n = 155%**	**Total n = 385%**	p-value*
**TMJ sounds**	14.3	12.3	13.5	Ns
**TMJ pain**	2.2	3.2	2.6	Ns
**Muscle tenderness**	0.4	0.6	0.5	Ns
**Restricted opening**	6.5	1.9	4.7	*
**Opening deviation**	6.1	0.6	3.9	***
**At least one sign**	25.2	15.5	21.3	*

ns: non significant

*p < 0.05

**p < 0.01

***p < 0.001

Restricted mouth opening, opening deviation and at least one sign of TMD were significantly more frequent in females than males. Opening deviation was 6.1% and 0.6% for females and males respectively. The amplitude of mouth opening, overbite taken into account was 46.5 mm and 50.2 mm for females (6.5%) and males (4.7%) respectively. Table 3 shows the percentage distribution of TMD symptoms according to gender. Thirty three percent of the whole sample reported at least one symptom with females significantly more than males. The most frequent symptom of TMD was headache (22%) while jaw locking was the least prevalent sign (2.1%). Generally the prevalence of symptoms was higher in females than males, however only pain during chewing showed a significant difference. From the questionnaire, the percentage distributions of some parafunctional habits are shown in Table 4. Lip/cheek biting was the most frequent habit (41%) and the females (45.8%) were significantly more than the males (15.6%) (p=.001). Nail biting was the second most frequent habit (29%) with no gender difference. Moreover, no statistical difference was found in bruxism (7.4%) or thumb sucking (7.8%) between males and females.

**Table 3:** Percentage distribution of TMD symptoms according to gender

**Symptoms of TMD**	**Females n = 230 (%)**	**Males n = 155 (%)**	**Total n = 385 (%)**	P-value*
**Headache**	24.5	12.1	22	Ns
**TMJ noise**	9.1	6.1	8.7	Ns
**Pain during chewing**	15.6	3.1	14	*
**Difficulty opening**	2.9	0	2.5	Ns
**Jaw locking**	1.9	3.1	2.1	Ns
**At least one symptom**	35.1	21.9	33	*

ns: non significant

*p < 0.05

**Table 4:** Percentage distribution of Parafunctional habits according to gender

**Parafunction**	**Females n = 230 (%)**	**Males n = 155 (%)**	**Total 385%**	P-Value*
**Lip/cheek biting**	45.8	15.6	41	**
**Nail biting**	28.5	33.3	29	ns
**Bruxism**	6.7	12.1	7.4	ns
**Thumb sucking**	9	0	7.8	ns

ns: non significant

** p < 0.001

## Discussion

A number of studies of the prevalence of TMD in children and adolescents have been published from different parts of the world, (Table 1).

The aim of this study was to evaluate the prevalence of signs and symptoms of TMD in adolescent school children through clinical examination and subjective data obtained from questionnaires and compare the findings with other national and international studies.

The present study has shown that the prevalence of clinical signs and symptoms was 21.3% and 33% respectively, with females statistically higher than males. These results are in agreement with similar results reported by Farsi [7] Nourallah [11] Thilander [23] Dibbet [24] and Abdulhakim [38]. Also, the present results were lower than some previous reported studies [17-19,25,30] while slightly higher than others [5,6,20-22]. The large frequency ranges for signs and symptoms of TMD previously described in reviews and meta-analysis are apparently based on very different samples (e.g. random vs. non-random, patient vs. non patient, different ages, age ranges, sample size, ratio of gender distribution) and different examination methods (e.g. kind of variable, method of data collection) [2]. It is tempting to believe that the wide range of differences in the prevalence of TMD is of racial origin [23], as in similar reports from Japanese [21,22] and Chinese populations [20], and similar reports from Swedish [25] and Finish [26] populations. However other reports do not support this theory and differences in the prevalence of TMD do exist not only between various populations but within samples of the same population and of the same age [23].

TMJ sounds are often an indication of mechanical interferences with the joint [27]. In the present study the most prevalent sign of TMD was TMJ sounds 13.5%, with no apparent gender difference. This is in agreement with reports by Egermark-Erikson (18), Ogura [21] and Widmalm [28]. Similarly, Farsi [6] in a sample of females only reported that 15% in the adolescent group had TMJ sounds, and in another study, she reported that males and females adolescents with permanent dentition had 12.8% TMJ sounds [7]. Some studies reported much higher incidence of TMJ sounds, but this was due to the use of stethoscope in their clinical examination [29-31]. Although TMJ sounds have been found to be significantly more common in girls than boys [7,18,32] this was not confirmed in this study nor in other previous reports [33,34]. It is interesting that clinical studies have reported a prevalence of TMJ sounds ranging from 8%–33%. Methods and criteria for recording joint sounds differ in the various reports, and thus, combined with natural fluctuations, is possibly the reason for the wide range of joint sounds.

Prevalence of Mandibular opening restriction was low (4.7%), yet there was a significant difference between males (1.9%) and females (6.5%). In the present study, mouth opening of less than 40 mm was considered as restricted opening as reported by Okeson [16]. The amplitude of mouth opening, overbite taken into account, reached 46.5 mm and 50.2 mm for females and males respectively. The results show that the average mouth opening is greater in males than in females. These results correspond to the average data published by Farsi [6], Grosfeld et al [29], Solberg et al [32], Mezitis et al [35] and Cox et al [36]. More recently Gallgher et al [37] reported almost similar results of 42.6 and 44.6 for females and males respectively. In this study it seems that restricted opening (4.7%) may occur without other accompanying signs of muscle tenderness which was only 0.5% or TMJ pain which was 2.6%. Some individuals may have restricted opening without pain or muscle tenderness. This is supported by studies on TMJ symptom free subjects [35-37] where the maximum opening range reached 33.7–60.4 in one study [35] and 38.7–67 in another [36]. Gallagher el al [37] further added that there was no differences in the maximum opening between normal and abnormal subjects (abnormal was defined as clicking or attending to a doctor because of trouble with the jaw joint).

Opening deviation in spite of its low occurrence (3.9%) was also found to be significantly more in females (6.1%) than males (0.6%). It seems that opening deviation movements in persons of the age group observed in this and other studies [6,7,29,37] appears rarely in epidemiological studies. Therefore reduced range of deviation movement can be regarded as an important sign or symptom in the diagnosis and treatment of TMD [29].

TMJ pain, 2.6%, and muscle tenderness, 0.5%, appear to be very low in the present study, similar results have been reported by Farsi [6], Ogura[21] and Kristinelli [31]. However higher prevalence was reported by others [30,34].

Clinical signs from TMJ in this study apart from sounds were low in occurrence, similar to other reports from different populations [7,18,20,33].

Reported symptoms from questioners revealed that 33% of the subjects had at least one symptom of TMD. Similar results were reported by Farsi [7], Thilander [24], Nilner [34] and Abdel-Hakim et al [38]. In the present study headache was the highest prevalent symptom, 22%. This is in agreement with other reports by Farsi [7], Nilner [17] and Widmalm et al [28], however Abdel-hakim et al [38] reported higher percentage of Saudi adolescents suffering of headache, 34%. Headache was reported to be significantly more in females [7,28,37], which was also found in the present study, however the difference was not significant. Since in this study restricted opening were low in occurrence, muscle tenderness and related pain and tenderness in the TMJ were considered rare, headache could not be only related to TMD symptoms. Headaches are common among children and adolescents particularly premenstrual headache, migraine, stress and tension- type headaches and headache due to high blood pressure [46]. Therefore reported headache could have other causes than overload of TMD muscles. Liljeström M-R et al studied the association of TMD and headache, among other problems, in a specific group of adolescents with primary headaches. They concluded that TMD should always be considered when headache is associated with earache, difficulties in opening the mouth, fatigue or stiffness of jaw and tenderness of masticatory muscles [47]. Other factors contributing to headache could be psychological factors such as anxiety and depression [48]. Bonjardim et al studied the prevalence of anxiety and depression in non patient adolescents and their relationship to signs and symptoms of TMD. They reported that anxiety (16.5%) and depression (26.7%) although of mild intensity, are common in adolescents. Their findings also show association between anxiety and depression and subjective symptoms [49].

Pain during chewing, 14%, was the next most common symptom and was found to be significantly more common in females (15.6%) than males (3.1%). Other symptoms such as hearing TMJ noise, difficulty opening the mouth and jaw locking were rare.

Reported parafunctional habits were not common in this study except for lip/cheek biting (41%) and nail biting (29%). Reports in the literature fluctuate, in Saudi adolescents; Abdel-Hakim reported 33% lip/cheek biting and only 16% nail biting [38] while Farsi reported 38% and 33% respectively [7]. Cheek biting was significantly more common in females (45%) than males (15.6%), and these results are comparable to Egermark-Eriksson et al [18] who reported combined nail and cheek biting to be 48% in their study group. Their method was similar to the present study where the children were assisted in answering the questionnaire. Higher results of nail or cheek biting habits (55%) were reported by Nilner and Kopp [39], and Wanman [40]. Other parafunction habits such as thumb sucking were low in occurrence, only in females (9%) and bruxism was 7.4% in the whole group.

Although some reports noted no sex differences in the prevalence of TMD [20,21,30], this has not been the case for some of the signs and symptoms in the present study. Generally females have more signs and symptoms than males. This is in agreement with other reports in the literature[7,24,32,33,41]. It has been stated that these sex differences could probably be explained by mental factors i.e. young females seem to present a lower pain threshold [41]. Other factors such as stress is well known from TMD studies in adults that women are more affected than men [4,9,41]. Sex difference may also be explained by some physiological changes seen at pubescence, as in the present study. The pattern of onset of TMD after puberty and lowered prevalence rates in the postmenopausal years suggest that female reproductive hormones may play an etiologic role in temporomandibular disorders [50]. This is also supported by the longitudinal data reported by Magnusson et al [51]. They found that gender difference in signs and symptoms was small in childhood, but from late adolescence females reported more symptoms and exhibited more clinical signs than males did.

A significant point to be learned is the need for thorough diagnosis and awareness by the orthodontics of potential TMD before of initiation of treatment. Therefore including an evaluation of TMJ, muscles and related mandibular function in routine dental examination in adolescents seems justifiable, to identify those who are a potential risk for TMD, especially before starting any orthodontic treatment. Further reports will investigate and assess correlation of TMD and occlusal characteristics.

## Conclusion

This report has primarily been a description of the clinical signs and symptoms of TMD and oral parafunctions in adolescents with special reference to gender differences. The prevalence of TMD signs were 21.3% with joint sounds being the most prevalent sign. While symptoms were found to be 33%, with headache being the most prevalent. Among the oral parafunctions, lip/cheek biting was the most prevalent 41% followed by nail biting 29%.

## Competing interests

The author(s) declares that she has no competing interests.
